# Risk of Hospitalization for Acute Cardiovascular Events among Subjects with Lower Urinary Tract Symptoms: A Nationwide Population-Based Study

**DOI:** 10.1371/journal.pone.0066661

**Published:** 2013-06-12

**Authors:** Huey-Juan Lin, Shih-Feng Weng, Chun-Ming Yang, Ming-Ping Wu

**Affiliations:** 1 Department of Neurology, Chi Mei Medical Center, Tainan, Taiwan; 2 Department of Medical Research, Chi Mei Medical Center, Tainan, Taiwan; 3 Division of Urogynecology, Department of Obstetrics and Gynecology, Chi Mei Medical Center, Tainan, Taiwan; 4 Department of Cosmetic Science, Chia Nan University of Pharmacy and Science, Tainan, Taiwan; 5 Center of General Education, Chia Nan University of Pharmacy and Science, Tainan, Taiwan; 6 Department of Hospital and Health Care Administration, Chia Nan University of Pharmacy and Science, Tainan, Taiwan; University of Sao Paulo Medical School, Brazil

## Abstract

**Background and Purpose:**

Lower urinary tract symptoms (LUTS) have been reported to be associated with metabolic syndrome and may predispose subjects to cardiovascular disease. The magnitude of the impact on the medical care remains to be elucidated. Based on a population-based claims dataset in Taiwan, we explored the association between LUTS and the risk of subsequent hospitalization for acute cardiovascular events.

**Materials and Methods:**

Among a representative sample of one million subjects from nationwide health insurance enrollees, subjects with codes of LUTS in service claims and without previous cardiovascular diseases including stroke were compared with age- and sex-matched non-LUTS subjects in subsequent hospitalization for acute coronary syndrome or stroke from the recruited date (between 2001–2004) to 2009. The risk of outcomes was assessed with Kaplan-Meier curves and the impact of LUTS was estimated with Poison regression analysis and Cox proportional hazards models.

**Results:**

We included 4,553 LUTS subjects and 22,765 matched non-LUTS subjects, with a mean age of 47 years and 43% of men. Hypertension, diabetes, and hyperlipidemia were more prevalent in the LUTS group. The incidence rate of the composite endpoint was significantly higher in the LUTS group than in the non-LUTS group (5.4/1000 vs. 4.0/1000 person-years). The difference mainly derived from stroke rather than acute coronary syndrome. After adjusting for age, sex, diabetes, hypertension, and hyperlipidemia in multivariable analysis, LUTS remained a significant predictor (hazard ratio, 1.29; 95% confidence incidence, 1.06–1.50).

**Conclusion:**

Subjects free of cardiovascular disease and presenting with LUTS are at risk of subsequent hospitalization for acute cardiovascular events, mainly stroke. The information might prompt practitioners encountering such patients to undergo appropriate diagnostic and preventive measures.

## Introduction

The high prevalence of Lower urinary tract symptoms (LUTS) but with a low healthcare seeking rate [1], and the potential negative impact of LUTS on overall health have caught global attention [2], [3]. It is noted that LUTS progress with only a minority regressing [4]. Some components of LUTS have been linked to metabolic syndrome [5], and thus carry common risk factors for cardiovascular disease. LUTS might precipitate the development of cardiovascular events through multifactorial interlacing processes such as autonomic nervous dysfunction [6], [7], affective disorders [8], or adverse effects of medications for treating LUTS [9], [10]. However, the magnitude of the LUTS burden on the medical care for cardiovascular disease in a population scale remains unclear.

Using the nationwide population-based health insurance claims data in Taiwan, we conducted a cohort study to test the hypothesis that LUTS might be associated with increased risk of subsequent hospitalization for acute coronary syndrome (ACS) or stroke.

## Materials and Methods

### Data source

The source of data is the National Health Insurance (NHI) Research Database (NHIRD) in Taiwan. By 2009 more than 99% of the total population in Taiwan has been covered by the compulsory and universal NHI since its implementation in March 1995. The details of NHIRD were described in our previous studies [1], [11]. In brief, from the registration files and NHI claims data between 2000 and 2009, a random sample of one million enrollees (approximately 5% of Taiwan's population) has been established as a representative cohort. There are no statistically significant differences between this cohort and the entire population in age, sex, or health care expenditures. The NHIRD provides information of encrypted patient identification numbers, sex, date of birth, dates of outpatient visits, the International Classification of Diseases-9th Revision-Clinical Modification (ICD-9–CM) codes of diagnoses and procedures, details of prescriptions and expenditure amounts, inpatient expenditures by admissions, etc. All NHI datasets can be interlinked through each individual personal identification number. In conformance to the Personal Information Protection Act, the unique identifiers of the subjects and the institutes have been scrambled cryptographically to assure anonymity. Confidentiality assurances are addressed by abiding by data regulations of the Bureau of NHI, and we have consulted with the Institutional Review Board of our hospital and obtained a formal written waiver for the need of ethics approval (No. 10202-E07).

### Definition of LUTS

In accordance with our study series on LUTS [1], [11], we scrutinized all the relevant ICD-9-CM codes and defined the following categories of codes for service claims as LUTS: (A) storage symptoms, including hypertonicity of bladder (ICD-9-CM code 596.51), frequency and polyuria (788.4), stress urinary incontinence in women (625.6) and men (788.32), urgent incontinence (788.31), nocturnal enuresis (788.36), nocturia (788.43), mixed incontinence (788.33); (B) voiding symptoms, including retention of urine (788.2), splitting & slowing of urine stream (788.6), and post-void dribbling (788.35); and (C) benign prostate hyperplasia (BPH) in men (600). Because LUTS are inseparable from clinical BPH [12], men with a BPH code, regardless of corresponding codes for storage/voiding symptoms or not, were all categorized as LUTS.

### Assembly of study subjects

We defined the recruitment period of 2001 to 2004, and identified men and women with either one of the following criteria as the LUTS group: (1) at least three outpatient service claims with the codes of LUTS at any clinics within one year after the first LUTS code, or (2) any one single hospitalization with LUTS among the 5 principal claims diagnosis codes. For each subject, the date of the first LUTS code was designated as the index date of entry. We excluded those subjects who had ICD-9 codes of coronary artery disease (410–414) or stroke (430–438) before the index entry date. From subjects without LUTS and free of cardiovascular diseases, a comparison group was assembled by matching a LUTS subject with 5 non-LUTS subjects on index entry date, sex, and age (±30 days).

### Follow-up and outcome measures

The primary outcome of interest was a composite endpoint of the first hospitalization for either ACS (a discharge ICD-9 code of 410 for acute myocardial infarction, or 411 for other acute and subacute forms of ischemic heart disease) or acute stroke (430–431 for hemorrhagic stroke, 433–434 for ischemic stroke, 435 for transient ischemic attack, or 436 for acute but ill-defined cerebrovascular disease). The secondary outcomes were hospitalizations for ACS and acute stroke separately. The subjects were followed up until death or the end of 2009. According to the regulations, enrollment of NHI is mandatory for all the population, and must be withdrawn within 30 days after decease. Since the National Death Registry was not available for our study, we determined the vital status by an indirect way. From the claims database, those subjects who withdrew the NHI enrollment within 30 days after discharge from the last hospitalization were presumed dead, and the discharge date was designated as the date of death.

### Statistical analysis

The demographic and clinical characteristics were compared between the LUTS group and the non-LUTS group. Age was classified into three categories: 18–39, 40–59, ≥60 years. To adjust for potential confounders for cardiovascular disease, we selected a set of comorbidities including hypertension (ICD-9 codes 401–405), diabetes (250), and hyperlipidemia (272), each of them as a dichotomous variable. Student t-test was used for continuous variables and Chi-square test for categorical variables. The incidence rate was calculated as the number of outcomes divided by the total person-years of follow-up. We assessed possible overdispersion of the count outcome data by testing whether the negative binomial dispersion parameter was significantly different from zero. Because it was significant, a Poisson regression model by SAS PROC GENMOD taking overdispersion into account was used to estimate the incidence rate ratio (IRR) between the two groups. We used Kaplan-Meier curves to plot the cumulative risk of hospitalization for the composite endpoint (ACS or stroke), and separately for ACS and for stroke, comparing the LUTS group and non-LUTS group with the log-rank test. Subjects with interim mortality were censored at the date of death. Cox proportional hazards models were used to estimate the effect of LUTS on the composite outcome by adjusting for potential vascular risk factors. Age, sex and the selected comorbidities have been shown to be risk factors for cardiovascular disease. We considered that there would be no problem of overfitting (the use of models that include more terms than are necessary) and decided to include all the variables simultaneously in the multivariable analysis. Since the pathogenesis of LUTS might vary between men and women due to anatomical differences and underlying diseases, we examined whether the effect of LUTS on outcome was modified by sex by including an interaction term in the multivariable model. A two-tailed *P* value of <0.05 was considered statistically significant. All the analyses were performed with the SAS software version 9.3 (SAS Institute, Cary, NC).

## Results

The study included 4,553 subjects with LUTS and matched 22,765 subjects without LUTS. The age and sex distributions were comparable, with a mean age of 47 years and 43% of men. Vascular risk factors were significantly more prevalent in the LUTS group than in the non-LUTS group (Table 1).

**Table 1:** Basic characteristics between the LUTS group and the non-LUTS group.

	LUTS (N = 4553)	Non-LUTS (N = 22765)	*P*-value
Age, mean ±SD, year	47±15	47±15	0.980
18∼40	1483(32.6)	7415(32.6)	1.000
>40∼60	2085(45.8)	10426(45.8)	
>60	985(21.6)	4924(21.6)	
Men	2467(43.0)	9790(43.0)	1.000
Comorbidities			
Diabetes	272(6.0)	799(3.5)	<0.001
Hypertension	471(10.3)	1405(6.2)	<0.001
Hyperlipidemia	157(3.5)	440(1.9)	<0.001

Data are number (%). LUTS =  lower urinary tract symptoms; SD =  standard deviation.

**Figure 1:**
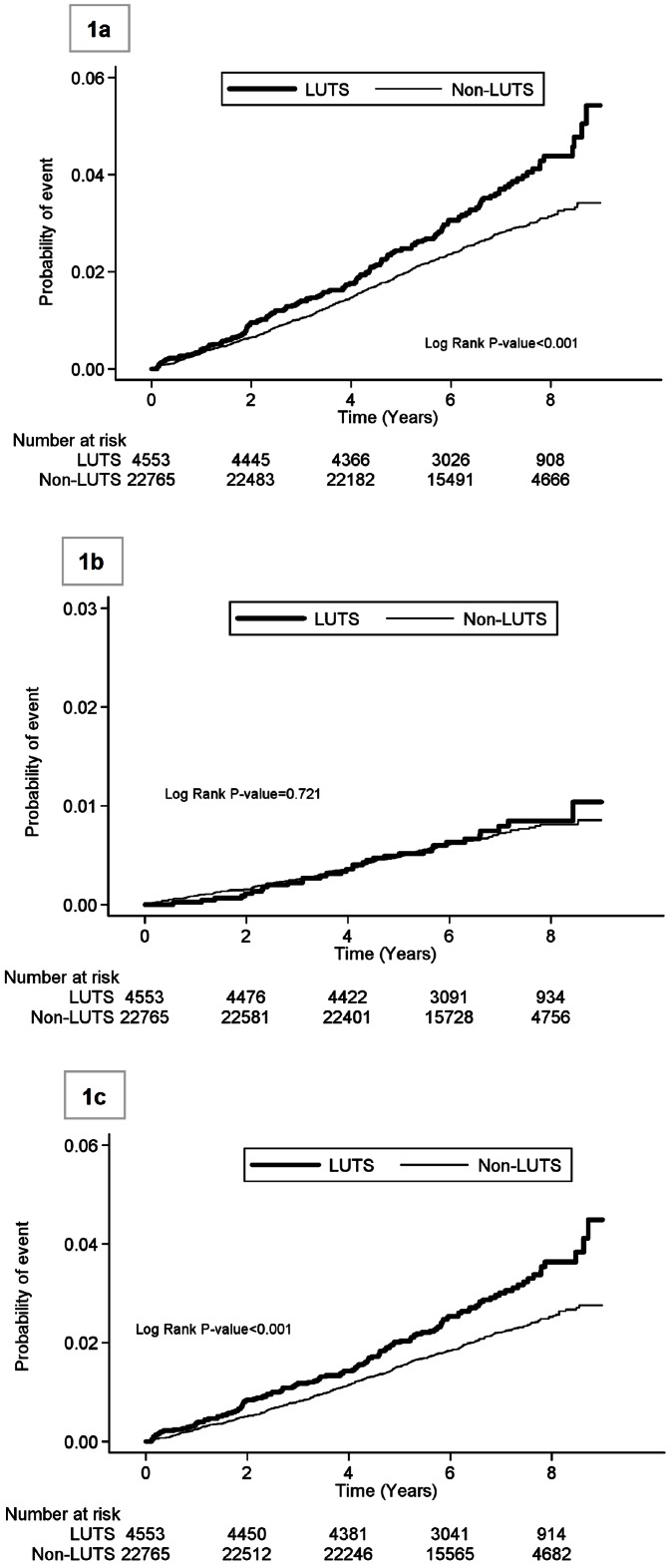
Probabilities of hospitalization for (1a) composite outcome of acute coronary syndrome or stroke, (1b) acute coronary syndrome, (1c) stroke, in subjects with and without lower urinary tract symptoms (LUTS).

The average follow-up duration was 6.6±1.5 years for the LUTS group and 6.7±1.4 years for the non-LUTS group. During the period, the composite outcome occurred in 162 (32 with ACS and 130 with stroke) of the LUTS group and 613 (138 with ACS and 475 with stroke) of the non-LUTS group. The distribution of stroke subtypes was similar, with 62% of infarction, 16% of transient ischemic attack, 17% of hemorrhage, and 5% of unspecified in the LUTS group, and 58% of infarction, 13% of transient ischemic attack, 24% of hemorrhage, and 5% of unspecified in the non-LUTS group (*P* = 0.319). The incidence rate of the composite endpoint was significantly higher in the LUTS group than in the non-LUTS group overall (5.36 vs. 4.00/1000 person-years, an increase of 34%, Table 2). Men appeared to have higher risks than women, but the differential magnitude between LUTS and non-LUTS groups was similar in both sexes. The incidence rate escalated as age increased, while the between-group difference manifested most in the subjects >60 years (Table 2). Kaplan-Meier survival curves show that the LUTS group was more likely to be hospitalized for a vascular event (9-year cumulative incidence, 5.2%; 95% confidence interval, 4.2–6.4%) than the non-LUST group (3.5%; 3.2–3.9%) (Figure 1A), and the difference mainly derived from hospitalization for stroke rather than for ACS (Figures 1B and 1C).

**Table 2:** Risks of hospitalization for the composite outcome (acute coronary syndrome or stroke) in the LUTS group and the non-LUTS group.

Characteristics	LUTS				non-LUTS					
	N	Composite outcome	PY	IR	N	Composite outcome	PY	IR	IRR* (95% CI)	*P* value
All	4553	162	30211	5.36	22765	613	153333	4.00	1.34 (1.13–1.59)	<0.001
Age, yr										
18∼40	1483	5	10152	0.49	7415	24	50974	0.47	1.05(0.40–2.74)	0.927
>40∼60	2085	50	13979	3.58	10426	218	70329	3.10	1.08(0.82–1.44)	0.361
>60	1630	107	6080	17.60	4924	371	32030	11.58	1.61(1.37–1.88)	<0.001
Sex										
Men	1958	105	12627	8.32	9790	623	80134	6.06	1.37(1.11–1.70)	0.004
Women	2595	57	17584	3.24	12975	222	88843	2.50	1.31(0.97–1.74)	0.080

LUTS =  lower urinary tract symptoms; PY =  person-year; IR =  incidence rate, per 1000 person-years; IRR =  incidence rate ratio.

* Estimated with Poisson regression.

In univariate Cox regression analyses, LUTS as well as older age, men, and vascular risk factors were significantly associated with increased risk of the composite outcome. After adjustment for all the other variables, LUTS remained statistically significant, increasing 29% risk of subsequent hospitalization for a vascular event (Table 3). No interaction between LUTS and sex was found (*P* = 0.889 for the interaction term).

**Table 3:** Univariate and multivariable Cox proportional hazards regression analyses for the risk of hospitalization for the composite outcome.

Variable	Crude HR (95% CI)	Adjusted* HR (95% CI)
LUTS	1.34(1.13–1.60)	1.29(1.06–1.50)
Age(per 10 years)	2.03(1.93–2.14)	1.86(1.76–1.97)
Men	2.46(2.13–2.85)	1.60(1.38–1.86)
Diabetes	5.39(4.46–6.51)	2.54(2.07–3.13)
Hypertension	4.28(3.62–5.06)	1.73(1.44–2.08)
Hyperlipidemia	2.62(1.91–3.59)	0.91(0.65–1.27)

* The model contained all the listed variables.

LUTS =  lower urinary tract symptom; HR = hazard ratio; CI = confidence interval.

## Discussion

In this population claims data-based cohort study, we found that after adjusting for age, sex, diabetes, hypertension, and hyperlipidemia, LUTS was associated with increased risk of subsequent hospitalization for ACS or stroke in subjects who were initially free of cardiovascular disease and stroke. This study had several strengths. The use of a population-based sample which has been verified in representativeness made the study findings generalizable to a national level. The follow-up cohort study design might eliminate major potential biases. We used hard endpoints (hospitalization for ACS or stroke) which have important clinical relevance.

Although LUTS were reported to be associated with vascular risk factors in previous research [13]–[15], there have been few large-scaled studies assessing the subsequent cardiovascular risks in subjects with LUTS. In a longitudinal study limited in men with an average follow-up of 6 years, Wehrberger et. al. reported that subjects with severe LUTS had an approximately 4-fold increased risk of coronary vascular disease and stroke compared with those with no or mild LUTS [16]. Our findings confirmed the increased risk of developing vascular events in subjects with LUTS but with a lower magnitude. It is notable that the 9-year cumulative incidences of vascular events in our subjects were much lower (5.2% in the LUTS group and 3.5% in the non-LUTS group) than those in the Wehrberger's cohort (4.4% in subjects with no or mild LUTS, and 29.4% in those with severe LUTS), although the average age of the study subjects appeared comparable. This might be explained by the differences of study designs. We included both men and women who had had no previous cardiovascular disease and thus overall might carry lower risk of incident events. Our subjects with LUTS encompassed all levels of severity, and those with mild LUTS might bear lower risk. Finally we counted only hospitalizations for vascular events and therefore might have left out the non-hospitalized ACS or stroke. Although men did have higher risk for vascular events than women, we found no interaction between sex and LUTS, and the risk magnitude associated with LUTS was similar between men and women (incidence rate ratio, 1.37 for men and 1.31 for women, Table 2).

As in Western countries, cardiovascular disease is the second leading cause of death and cerebrovascular disease the third in Taiwan. According to the official national annual health statistics reports (http://www.doh.gov.tw/CHT2006/DM/DM2_2_p02.aspx?class_no=440&now_fod_list_no=11468&level_no=1&doc_no=77184), hospitalizations for cardiovascular disease are less often than those for cerebrovascular disease, although the population died more of cardiovascular disease than of cerebrovascular disease. In our study outcomes which were limited to acute vascular events, hospitalizations for stroke apparently outnumbered those for ACS in both LUTS and non-LUTS groups. This finding was consistent with the national trends.

There have been proposed mechanisms to explain the association between LUTS and cardiovascular disease. In men, the finding that LUTS was associated with erectile dysfunction might imply a common pathogenesis of endothelial dysfunction contributing to cardiovascular morbidity [17], [18]. LUTS might result in sleep disorder which could cause adverse hemodynamic effects and predispose the subjects to vascular events [13]. Theoretically these mechanisms might affect both cardiovascular and cerebrovascular systems. However, it was intriguing that our subjects with LUTS were at risk of hospitalization for stroke but not for ACS. A plausible explanation might be due to the adverse central nervous system effects of medications used to treat LUTS. Antimuscarinic agents, often prescribed for subjects with LUTS, may have cognitive and cardiovascular effects [19]. On the other hands, it has been noted that a higher proportion of the treated patients with overactive bladder symptoms had pre-existing syncope and dizziness than untreated patients [10]. The medications for LUTS might exert untoward effects on central nervous system and hemodynamic status, in particular for patients with brain comorbidities. Currently we were not able to verify the underlying causes due to the study design and limited information. Nevertheless, our study implies that in further assessing the relationship between LUTS and vascular events, it might be necessary to discern ACS and stroke separately.

We developed a multivariable model to predict a predetermined composite outcome including ACS and stroke. As predicted, increasing age, men, diabetes, and hypertension were associated with subsequent hospitalization for acute vascular events. On the other hand, hyperlipidemia was statistically not a significant predictor. In the sub-analyses of evaluating these risk factors separately for ACS and for stroke, age, sex, diabetes and hypertension remained significant, while hyperlipidemia was non-significant for both outcomes (data not shown). The seemingly protective effect (hazard ratio  = 0.91) of hyperlipidemia might be purely due to chance, or derive from treatment with statin. Since the study was based on claims data, it was very likely that subjects with an ICD code for hyperlipidemia were treated with lipid lowering drugs including statin. Since the medication details were unavailable, we were unable to confirm this speculation.

Our study had several limitations, some of which were inherent in research using administrative data. First, the diagnosis codes (ICD-9-CM) are potentially problematic. We had no information regarding the accuracy of the codes for LUTS. Since LUTS are mostly symptom diagnoses, it was likely that LUTS were under-coded than over-coded. Our previous study using the same claims data reported that the healthcare-seeking prevalence for LUTS was 38 per thousand in 2009 [1], which might represent only a portion of people truly with LUTS. This type of systematic misclassification might have led to an underestimate of the LUTS effect on the outcomes. Second, some other important vascular risk factors such as obesity and cigarette smoking were not available and thus potential confounding effects might exist. Third, documented vital status was not accessible and our tactic in determining interim mortality and the date of death might not be accurate. However, as participation in the NHI program is mandatory and the coverage provided by NHI very comprehensive, the errors might be minor and would not substantially alter our main findings. Although with these limitations, this study might serve as a pilot for exploratory purposes. Since LUTS are highly prevalent, even a modest association with increased risk of significant morbidities would be worthy of attention in clinical practice. Several issues remain to be clarified in further research. It might be warranted to conduct prospective follow-up studies containing details of covariates, stratifying LUTS severity, and elucidating the differential effects of individual LUTS components on solid health outcomes.

In conclusion, for subjects without prior cardiovascular disease and stroke, LUTS may be associated with a modest but significant risk of subsequent hospitalization for acute vascular events, mainly stroke. This information might alert clinical practitioners treating these common symptoms to undergo appropriate diagnostic and preventive measures.
